# Historic evidence to support a causal relationship between spirochetal infections and Alzheimer’s disease

**DOI:** 10.3389/fnagi.2015.00046

**Published:** 2015-04-16

**Authors:** Judith Miklossy

**Affiliations:** Prevention Alzheimer International Foundation, International Alzheimer Research CenterMartigny-Croix, Switzerland

**Keywords:** Alzheimer’s disease, Borrelia burgdorferi, dementia, general paresis, oral spirochetes, Treponema spirochetes, Treponema pallidum, syphilis

## Abstract

Following previous observations a statistically significant association between various types of spirochetes and Alzheimer’s disease (AD) fulfilled Hill’s criteria in favor of a causal relationship. If spirochetal infections can indeed cause AD, the pathological and biological hallmarks of AD should also occur in syphilitic dementia. To answer this question, observations and illustrations on the detection of spirochetes in the atrophic form of general paresis, which is known to be associated with slowly progressive dementia, were reviewed and compared with the characteristic pathology of AD. Historic observations and illustrations published in the first half of the 20th Century indeed confirm that the pathological hallmarks, which define AD, are also present in syphilitic dementia. Cortical spirochetal colonies are made up by innumerable tightly spiraled *Treponema pallidum* spirochetes, which are morphologically indistinguishable from senile plaques, using conventional light microscopy. Local brain amyloidosis also occurs in general paresis and, as in AD, corresponds to amyloid beta. These historic observations enable us to conclude that chronic spirochetal infections can cause dementia and reproduce the defining hallmarks of AD. They represent further evidence in support a causal relationship between various spirochetal infections and AD. They also indicate that local invasion of the brain by these helically shaped bacteria reproduce the filamentous pathology characteristic of AD. Chronic infection by spirochetes, and co-infection with other bacteria and viruses should be included in our current view on the etiology of AD. Prompt action is needed as AD might be prevented.

## Introduction

Alzheimer’s disease (AD) is the most frequent cause of dementia. All efforts made in AD research during the last four decades provided important insights into the pathogenesis of AD but the cause of the disease is still unclear and the treatment unresolved.

The clinical manifestations of AD begin with subtle short-term memory deficit and anxio-depressive symptoms followed by orientation and language difficulties. The intellectual functions progressively disappear and the patients become entirely dependent. They may survive in this devastating state for more than a decade. Death generally occurs from secondary infection, frequently from pneumonia or urinary infection. The duration of the disease from the appearance of the first symptoms and the manifestation of dementia varies between 5 and 20 years.

The fact that AD usually develops in later life suggested that a slow-acting unconventional infectious agent acquired at an early age and requiring decades to become active may be involved in its etiology (Wisniewski, 1978; Khachaturian, 1985). Spirochetes are such unconventional infectious agents. In the long-standing, stationary or atrophic form of general paresis, *Treponema pallidum* (*T. pallidum*) persists in the brain, sustains chronic infection and inflammation and causes slowly progressive dementia. It is established that *T. pallidum* is responsible for the various neuropsychiatric manifestations of chronic neurosyphilis. Dementia develops years or decades following the primary syphilitic infection (Merritt et al., 1946). As spirochetes are strongly neurotropic, a series of investigations were undertaken showing that following a long latent stage various types of spirochetes in similar way to *T. pallidum* can cause dementia (Miklossy, 1993, 1994; Miklossy et al., 1994, 1995). It is noteworthy, that the human oral cavity harbors more than 60 different Treponema species (Dewhirst et al., 2000). These were previously considered as commensal spirochetes, but several of them revealed to be predominant and invasive periodontal pathogens (Riviere et al., 1991; Miklossy, 2011b). *T. pectinovorum, T. amylovorum, T. lecithinolyticum, T. maltophilum, T. medium* and* T. socranskii* were all detected in the brains of AD patients (Riviere et al., 2002). Another spirochete, *Borrelia burgdorferi (B. burgorferi)*, the causative agent of Lyme disease transmitted by infected tick bites to human (Burgdorfer et al., 1982), was also detected in the brain in a percentage of AD patients. The similarities of the clinical and pathological manifestations of syphilis and Lyme disease are well documented (e.g., Fallon and Nields, 1994). MacDonald and Miranda (1987) first reported the occurrence of *B. burgdorferi* in the brain of an AD patient. This was later confirmed by the same and by various other authors (MacDonald, 1988, 2006; Miklossy, 1993; Riviere et al., 2002; Miklossy et al., 2004). Exposure of primary neuronal and glial cells and brain cell aggregates to spirochetes induces plaque-, tangle- and granulovacuolar degeneration-like lesions, similar to those occurring in AD (Miklossy et al., 2006a). Spirochete specific antigens and DNA were co-localized with amyloid beta (Aβ). Several recent reviews concluded that there is a significant association between spirochetal infection and AD (Honjo et al., 2009; Miklossy, 2011a,b; De Chiara et al., 2012; Hill et al., 2014; Maheshwari and Eslick, 2015). That this strong association fulfills Hill’s nine criteria represented evidence in favor of a causal relationship (Miklossy, 2011b).

If AD is indeed caused by various chronic spirochetal infections, one should find AD-type lesions in syphilitic dementia as well. Therefore, the goal of the present study was to revisit the historic literature on the detection of spirochetes in the atrophic form of general paresis, which is known to be associated with slowly progressive dementia. The aim was to confirm that *T. pallidum* the causative agent of syphilitic dementia also reproduces the pathological hallmarks of AD. It is generally acknowledged that it is the invasion, the persistence and the accumulation of *T. pallidum* in the brain, which causes dementia in syphilis. Therefore, observations and illustrations on the detection of spirochetes in syphilitic dementia were compared to the characteristic pathology of AD. In order to ensure that the information presented is compliant to historic descriptions, particularly for strongly relevant subjects, citations from the original text or from published translations were sometimes used. Historic observations and illustrations indeed testify that the pathological hallmarks of AD also occur in the atrophic form of general paresis and indicate that persisting spirochetal infection can reproduce the clinical and pathological hallmarks of AD. These historic observations provide evidence in support of a causal relationship between various spirochetal infections and AD.

## Similarities of the Clinical Manifestations of AD and the Atrophic Form of General Paresis

Similarities between the clinical manifestation of AD and syphilitic dementia have long been observed (Hübner, 1909; Perusini, 1910; Alzheimer, 1911). Alzheimer (1911) himself noticed “how difficult it is to define diseases solely with respect to their clinical features”. Describing his second famous case (Johann F, 56-year-old) in 1911, Alzheimer stated that “the diagnosis of an atypical Lissauer’s paralysis could not, however, be dismissed until the death, largely because of an experience which we had only a few months before. A patient, who had shown quite a similar picture in many respects (profound mental impairment, sensory aphasia, agnosia and apraxia), and who, just like case F; had no abnormality of complement in either blood or cerebral spinal fluid on repeated examinations, the microscopic examination turned out to have a progressive general paralysis with atypical localization”.

Perusini (1910) analyzed the brains of four cases with presenile dementia given to him by Alzheimer, including the first famous case of Alzheimer (1907), Auguste Deter (case I: D. A. 51 years old female) and three other patients: case II: R. M, 45 years old male; case III: B. A., 65 years old female; case IV: Schl. L., 63 years old male). Perusini analyzed and compared the neuropathological findings of these cases and published his results in 1910. The alterations in all four cases were similar and limited to the cerebral cortex and consisted of tangles and plaques. Case IV (Schl. L) had a 30 years history of syphilis. Perusini concluded that the most striking similar alterations, neurofibrillary tangles and plaques might also be present in old cases of syphilitic dementia. He stated that “It is difficult, on the present basis of our clinical knowledge, to include or exclude these cases as syphilis. This has been made more difficult because case IV demonstrates a direct relationship with syphilis”.

Bonfiglio (1908) also analyzed the brain of a case provided to him by Alzheimer (Leonhard Sch., age 60, from Landshut (Bavaria), admitted to the Munich asylum on June 20th, 1904). He mentions, “Nothing is known about the clinical history of this patient.” However, the initials of the patient, the dates of hospitalization and death (1st January 1904) indicate that this patient is identical to case IV (Schl. L) examined by Perusini (1910), who suffered for 30 years from syphilis. As the chief pathological changes, Bonfiglio (1908) also describes the occurrence of fibrillary alterations of neurons and the miliary foci of necrosis similar to those of Alzheimer’s first case Auguste Deter. Bonfiglio (1908) stated: “I must mention that I had found these same foci even in other cases given to me by Alzheimer, cases which, as far as I know, have only one common feature, that is a history of syphilis”. "Indeed, many of the alterations observed in my own cases are similar to those found in certain forms of neurosyphilis. “I think the coincidence of this foci with the neurofibrillary alterations described above cannot be disregarded”.

The similarity of clinical symptoms between senile dementia and severe general paresis was also highlighted by Hübner (1909). Many other historic and recent observations underline the similarity of the clinical symptoms in syphilitic and AD-type dementia (Merritt et al., 1946; van Eijsden et al., 2008; Wang et al., 2011; Mehrabian et al., 2012). For many decades, syphilitic dementia was considered in the differential diagnosis of AD.

## Pathological Hallmarks Defining AD

The most characteristic pathological hallmarks of AD consist of the accumulation of senile plaques and neurofibrillary tangles and the deposition of beta amyloid in the brain. For the definite diagnosis of AD histological confirmation of these pathological changes are necessary.

It was Simchowicz (1911) who introduced the term “senile plaque” but Blocq and Marinesco (1892) observed plaques first in an elderly epileptic patient. Redlich (1898) described them in the case of a 78-year-old woman suffering from senile dementia as “miliary sclerosis” or miliary cortical plaques and discussed his similar findings in other cases analyzed.

Alzheimer (1907) observed cortical plaques and neurofibrillary tangles in the brain of Auguste Deter, a 51-year-old female patient, who suffered from presenile dementia, using the Bielschowsky (1904) silver technique. He published additional observations concerning this case in 1911, together with the clinical and pathological findings of another patient, Johann E. (J. E.), who had “plaque only” AD. He described senile plaques as miliary foci of deposits of a peculiar substance, which consist of a central core and a peripheral halo. He found that the plaques were numerous in the frontal, temporal and parietal cortex, were scarce in the central gyrus and less numerous in the occipital cortex. He found them in the striatum, lentiform nucleus and thalamus and in individual lobuli of the cerebellum. Some plaques were also visible in the gray matter areas of the pons and medulla oblongata. He described the laminar distribution of senile plaques in the cerebral cortex: “…They tended to accumulate in the 2nd and 3rd layers, were rarer in deeper layers…”. He first described neurofibrillary tangles, which, were called for a long time “Alzheimer’s fibrillary degeneration”. Following his original description (Alzheimer, 1907) “…inside an apparently normal-looking cell, one or more single fibers could be observed that became prominent through their striking thickness and specific impregnability. At a more advanced stage, many fibrils arranged parallel showed the same changes. Then they accumulated forming dense bundles and gradually advanced to the surface of the cell. Eventually, the nucleus and cytoplasm disappeared, and only a tangled bundle of fibrils indicated the site where once the neuron had been located”.

Fischer published his detailed analysis of senile plaques in 1907. He designated them as miliary plaque-like necrosis. He described senile plaque as a necrotic central focus surrounded by an area of oval or fusiform radiating formation. He found these miliary foci in 12 out of 16 cases of senile dementia but he did not find them in 10 normal subjects, in 45 cases of progressive paralysis, and in 10 cases of functional psychosis (Fischer, 1907). In 1910, Fischer also described the co-occurrence of senile plaques with neurofibrillary tangles in 17% of senile dementia cases. He noticed, “…the smallest plaques often show an apparent filamentous structure and reminds one of a bacterial colony.” The central part of the necrosis is similar to bacterial necrosis but do not cause inflammation. Gram and Ziehl-Neelsen techniques were negative but “the foci colored very easily with methylene blue … though no particular bacterial structure could be ascertained”. Fischer’s view was considered by several of his colleagues (e.g., Achúcarro, 1909; Perusini, 1910 etc.) Alzheimer (1911) also mentions Fischer’s view in his discussion on the origin of senile plaques noticing that “Fischer, pointed out their similarity to bacterial colonies and reported that he had undertaken cultivation experiments and complement fixation tests, which however produced negative results”. Alzheimer acknowledged Fischer’s detailed description of senile plaques and noticed “it will certainly remain to Fischer’s credit that he was the first to emphasize importance of the plaques to the histological appearance of senile dementia”. Fischer (1907), similarly to Alzheimer (1911) thought that these miliary foci consist of a peculiar foreign substance of unidentified chemical composition. Fischer supposed that the fibrillary proliferation in neurons formed *de novo* and the constituent material was unrelated to neurofibrils. In contrast, Alzheimer believed that the tangles consist of chemically modified neurofibrils.

Hübner (1909) described senile plaques as central accumulation of dark brown masses, which often demonstrate a star formation when impregnated with silver nitrate, with an increased staining of the surrounding tissue. Perusini (1910) described cortical deposition of “characteristic metabolic products as plaques”, fibrillar alteration of ganglion cells, astrocytic proliferation and formation of numerous rod-like glia (microgliosis). He observed that the apparently condensed fibrils are made up by numerous thin fibrils and thought, as Alzheimer, that the agglomeration of thin fibrils leads to sick fibrils (1910).

Neuropil threads or curly fibers were recognized very early, but it was from the discovery of the selective Gallyas (1971) silver technique, which selectively detect neurofibrillary tangles and curly fibers and from the introduction of immunohistochemical detection of tau that these pathological filamentous lesions were recognized as important contributors of AD pathology. It was Simchowicz (1911) who first described granulovacular degeneration, an intracytoplasmic neuronal alteration, which is also characteristic of AD.

With respect to local amyloid deposits, Oppenheim (1909) described “drusige Nekrosen” next to hyalinized blood vessels in about 50% of senile dementia cases and suspected that the material deposited in capillaries and senile plaques (“Drusen”) might be the same. Fischer (1910) provided the first illustration of “cerebral amyloid angiopathy” and Divry (1927, 1934), applying Congo red stain, determined that the homogenous deposit in senile plaque corresponds to amyloid (see also Berchtold and Cotman, 1998). Scholz (1938) described the occurrence of amyloid deposits in the wall of cortical and leptomeningeal vessels as “drusige Entartung” plaque-like degeneration—and it was Pantelakis (1954), who introduced the term congophilic angiopathy. Recent observations showed that the major subunit of fibrillary amyloid deposits is a small 4–4.2-kDa peptide (Aβ), which derives by proteolytic cleavage from a larger transmembrane amyloid beta precursor protein (AβPP; Glenner and Wong, 1984; Kang et al., 1987). Ultrastructural analyses showed that paired helical filaments are the main component of neurofibrillary tangles (Kidd, 1963; Terry, 1963; Terry et al., 1964). Further studies revealed that the microtubule associated protein tau (Brion et al., 1985a,b) is the major constituent of neurofibrillary tangles and curly fibers. It is present in an abnormally phosphorylated state (Grundke-Iqbal et al., 1986; Ihara et al., 1986) inhibiting microtubule assembly (Goedert et al., 1996).

## Pathological Hallmarks of the Atrophic Form of General Paresis

The atrophic form of general paresis is associated with a slowly progressive dementia caused by persistent *T. pallidum* infection. The interval from infection to onset of dementia varies between few months to 50 years, with an average of 20 years. In this form of general paresis, lymphoplasmocytic infiltrates are lacking or are rare and the number of spirochetes can be very high (Jahnel, 1916, 1917a,b,c, 1918, 1919, 1920, 1921, 1922; Pacheco e Silva, 1926, 1926–1927; Rizzo, 1931; Merritt et al., 1946; Schlossberger and Brandis, 1958; Miklossy, 2008a,b, 2011a). The number of spirochetes in the brain increases with the severity of dementia and the degree of brain atrophy (Pacheco e Silva, 1926–1927). In 1906, in a case of dementia paralytica, Sträussler (1906) described peculiar multiple “miliary areas of necrosis” in the cerebral cortex.

Noguchi and Moore (1913) detected *T. pallidum* spirochetes in the cerebral cortex of general paretic cases in 1913, showing, that these spirochetes can persist in the brain and cause the tertiary manifestations of neurosyphilis, including dementia. Jahnel (1916), introducing his modified silver technique for the visualization of spirochetes, analyzed their distribution in the brain in general paresis (Jahnel, 1916, 1917a,b,c, 1918, 1919, 1920, 1921, 1922). He described the dissemination of microorganisms scattered through the cerebral cortex in the form of circumscribed colonies or thick masses collected around cortical blood vessels (Jahnel, 1918, 1920). He noticed that the spirochetes are most numerous in the frontal cortex but may also occur in the basal ganglia and cerebellum. He also pointed out that their microscopic location was most prominent in the middle layers of the cerebral cortex. Later, Forster and Tomasczewski, 1913; Levaditi et al., 1913; Marie et al., 1913; Moore, 1913; Bouman, 1918; Hauptmann, 1919, 1920; Herschmann, 1920; Sprenger, 1920; Bravetta, 1921; Coppola, 1922; Manouélian, 1922; Schob, 1925; Pacheco e Silva, 1926, 1926–1927; Dieterle, 1928; Aars, 1930; Rizzo, 1931; Steiner, 1940; Merritt et al., 1946; Schlossberger and Brandis, 1958; Miklossy, 2008a; Miklossy et al., 2008c, and many others reported diffusely disseminated individual or plaque-like colonies of spirochetes in the cerebral cortex in general paresis.

Pacheco e Silva (1926, 1926–1927) noticed that the proliferation of spirochetes in the form of colonies or plaques in the cerebral cortex was particularly associated with the atrophic form of general paralysis. He studied the brains of more than 60 patients suffering from this atrophic form associated with progressive syphilitic dementia and found innumerable spirochetes in the cerebral cortex, in the basal ganglia, and in a case of tabo-paralysis in the pons and medulla oblongata. He mentioned the difficulty of finding spirochetes in the cerebellum.

Dieterle (1928), in addition to the dissemination of individual spirochetes, reported black patches of spirochetal colonies located in the gray substance. In many instances these foci dotted the entire convolutional arch (cerebral cortex), and perivascular or pericapillar colonies were often seen. He described countless spirochetal masses in the middle zone of the cerebral cortex and observed smaller subpial colonies. He noticed the absence of organisms within the white substance. Finally, he outlined the importance of the detection of spirochetes in the brain in general paresis. “It is the one type of histopathological examination that will reveal the true nature and extent of the infectious process”.

Steiner (1940) noticed that spirochetal conglomerations together with diffusely distributed spirochetes are usually present in a large number in the cerebral cortex or other gray matter areas. Morphologically, these ball-like masses are round or oval accumulations of spirochetes closely packed together. With silver-stains a characteristic feature of the central part of the mass consists of a compact yellow or brownish material, which, at high magnification, is formed by fine, lightly stained spirochetal threads that are “infinitely tangled”. At the periphery, the spirochetes are arranged in ray like strands, the axes of which radiate from the center forming a stellate pattern. The rays of this formation are made up of well-defined black spirochetes.

Cortical atrophy, neuronal loss, fibrillary alteration of neurons and severe astro- and microglia proliferation are also characteristic pathological lesions of the atrophic form of general paresis. Bick (1993) noticed that Alzheimer (1898) had found neurofibrillary alteration in the cerebellum in a case of progressive paralysis. The occurrence of neurofibrillary tangles in neurosyphilis was repeatedly documented (Bonfiglio, 1908; Perusini, 1910; Storm-Mathisen, 1978). Notably, argyrophilic granular form of spirochetes was also described in general paresis by several authors (Pacheco e Silva, 1926–1927).

Alzheimer (1897) described homogeneous colloid degeneration in the brain in general paresis, which was identified as local amyloidosis by Mignot and Marchand (1911) and later by Volland (1938) between many others. Recent characterization defined that the amyloid deposit in general paresis, as in AD, corresponds to Aβ (Miklossy et al., 2006b).

## Pathological Hallmarks of Syphilitic Dementia Compared to AD

**Figure 1:**
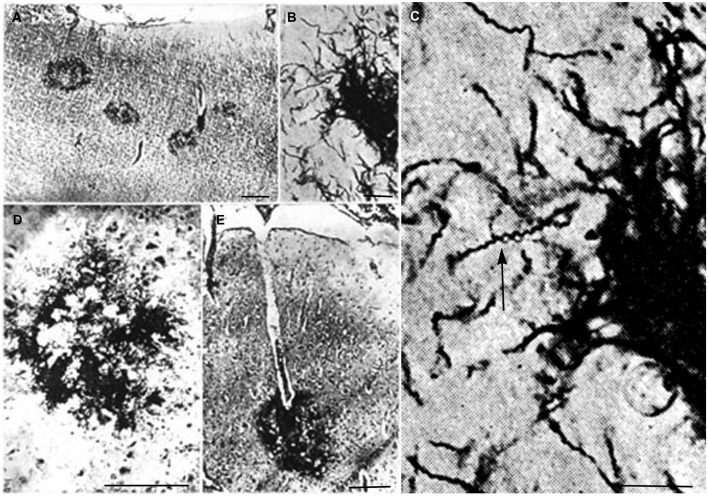
**Reproduction of illustrations published by Pacheco e Silva (1926–1927) showing plaque-like spirochetal colonies in the cerebral cortex of patients suffering from the atrophic form of general paresis. (A)** Argyrophilic spirochetal colonies are visible in the parietal cortex, morphologically similar to senile plaques. The legend used by the author himself: “Colonias de espirochetas em torno dos capillares periphericos do cerebro. Lobo parietal. Caso de paralysia geral. Meth. Jahnel. Pequeno augmento.” **(B)** At higher magnification the colonies are made up by individual spirochetes. **(C)** Further magnification of part of panel **(B)** showing the typical spiral appearance (arrow) of *T. pallidum* spirochetes. **(D,E)** Cortical spirochetal colonies morphologically undistinguishable from argyrophilic immature and perivascular senile plaques. These illustrations were reproduced from the original Figures 14A; 11B, C; 5/IID and 7E of Pacheco e Silva (1926–1927).

In advanced stages of the atrophic form of general paresis numerous spirochetes accumulate in the cerebral cortex. Their number increases with the severity of dementia and cortical atrophy (Jahnel, 1917a,b,c, 1918, 1919, 1920, 1922; Pacheco e Silva, 1926, 1926–1927; Rizzo, 1931; Merritt et al., 1946). They form balls, masses, plaques or colonies and/or disseminate as individual filaments in the cerebral cortex. Illustrations published in the first half of the last century show such accumulation of *T. pallidum* in masses or colonies in the cerebral cortex. Pacheco e Silva (1926, 1926–1927) who had analyzed the brain of more than 60 patients suffering from the atrophic form of general paresis illustrated spirochetal colonies or “plaques” in the cerebral cortex as shown in Figure 1. They are restricted to the cerebral cortex (Figure 1A). High magnification of Figure 1B, as seen in Figure 1C, shows the typical spiral morphology of *T. pallidum* (arrow) indicating that these argyrophilic “plaques” are indeed made up by spirochetes. The spirochetal colonies are restricted to the cerebral cortex and morphologically are undistinguishable from immature and perivascular senile plaques (Figures 1D,E, respectively).

**Figure 2:**
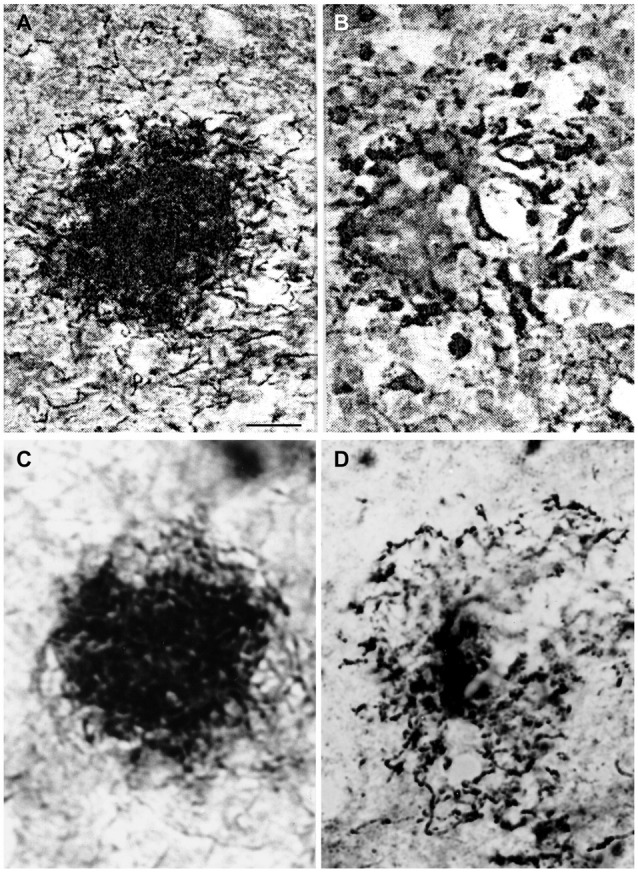
**Comparison of the morphology of senile plaques and spirochetal colonies in general paresis. (A,B)** Early **(A)** and degenerated **(B)** spirochetal colonies in the atrophic form of general paresis. **(C,D)**: Immature **(C)** and mature **(D)** senile plaques in Alzheimer’s disease (AD). Cortical paraffin sections of an AD case stained with Bosma-Steiner silver impregnation technique for the detection of spirochetes. Spirochetal colonies in **(A,B)** show the same morphological features as senile plaques in **(C,D)**. Panels **(A,B)** were reproduced from Figure 1 of Steiner (1940) who noticed with respect to **(A)** “Note the spread of spirochetes from the center and the peripheral liquefaction of tissue” and with respect to **(B)**: “A yellow center is shown, and the peripheral zones consist of a black ring with degenerating spirochetes and granules of spirochetal debris.” Permission for the reproduction was kindly provided by the American Medical Association (Copyright 1940). Bar: is the same for **(A–D)** and corresponds to 40 μm.

The identical morphology of spirochetal colonies and senile plaques is even more apparent when historic illustrations of spirochetal colonies are compared to silver impregnated senile plaques in AD. An even more selective staining of senile plaques and tangles can be obtained using silver impregnation techniques described for the visualization of spirochetes compared to the Bielschowsky (1904) or the modified Bielschowsky techniques (Bolle et al., 1992) routinely used in AD. When using the Bielschowsky technique for the visualization of AD-type changes, silver impregnation of several nerve fibers and glial cells also occur. Figure 2 compares the accumulation of spirochetes in the cerebral cortex in general paresis as illustrated by Steiner (1940) with senile plaques in AD, which were silver impregnated for spirochetes. The striking similarity of spirochetal and senile plaques is clear-cut. Compare the early (Figure 2A) and old, degenerated spirochetal colonies (Figure 2B), as illustrated by Steiner (1940) in general paresis, with the immature (Figure 2D) and mature senile plaques (Figure 2D) of AD.

**Figure 3:**
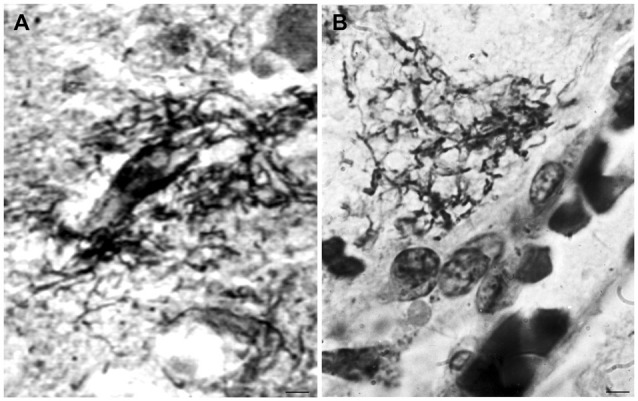
**Morphological similarity of perivascular spirochetal accumulation in general paresis with a small perivascular plaque in the cerebral cortex in AD**. Compare **(A)**, showing perivascular accumulation of spirochetes in general paresis with **(B)** where an amorphous perivascular cortical plaque is illustrated in AD stained with Bosma-Steiner silver technique. Panel **(A)** is reproduced from Figure 4 of Hauptmann (1920). Bar: 5 μm.

Cortical spirochetal colonies are not only similar to immature and mature senile plaques, but spirochetes can also form less dense masses morphologically identical to amorphous plaques. Accumulation of spirochetes around cortical vessels and capillaries is frequent (Figure 3). See the morphological similarity of the accumulation of spirochetes around a blood vessel in general paresis (Figure 3A) with that of a perivascular amorphous plaque in AD (Figure 3B).

**Figure 4:**
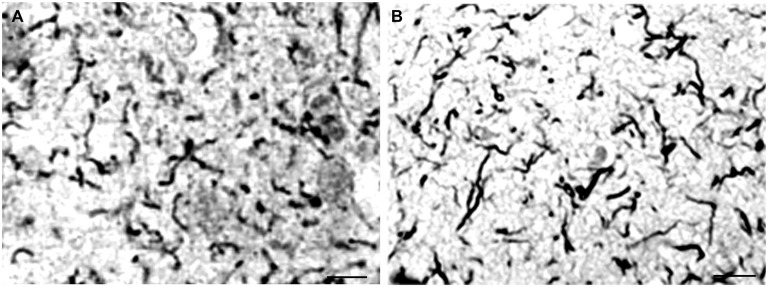
**Disseminated form of cortical spirochetosis in general paresis showing striking similarity to disseminated curly fibers in AD**. Compare **(A)**, where each filament corresponds to individual *Treponema pallidum* spirochete in general paresis, with **(B)**, where Gallyas silver impregnation technique shows disseminated curly fibers in the frontal cortex of an AD patient. **(A)** is reproduction of part of Figure 6 of Hauptmann (1920). Bars: 15 μm.

When the cortical dissemination of individual spirochetes in general paresis is compared with the distribution of curly fibers or neuropil threads in AD the similarity is striking. Figure 4 illustrates the distribution of individual spirochetes in general paresis and those of curly fibers in AD. Compare Figure 4A, where each filament corresponds to individual *T. pallidum* spirochete with disseminated curly fibers in AD (Figure 4B).

**Figure 5:**
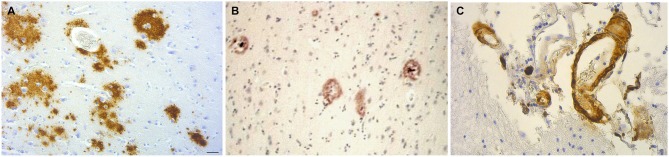
**The brain amyloid deposits in general paresis, as in AD, corresponds to beta amyloid. (A)** Cortical amyloid deposits in the brain in syphilitic dementia showing positive immunoreaction with anti-Aβ 8–17 (6F/3D, DakoCytomation) antibody. **(B)** Beta amyloid deposition similar to immature and mature plaques was observed. **(C)** Beta amyloid deposits in the wall of leptomeningeal arteries in the same case as **(A)**. For the immunohistochemical analysis of Aβ, the avidin-biotine-peroxidase technique was used and the paraffin sections were pre-treated with 80% formic acid for 20 min. Bar: 50 μm. Panels **(A)** and **(C)** were reproduced form Figure 2 of Miklossy et al. (2006b).

Local amyloidosis is known to occur in the atrophic form of general paresis. In order to show the similarity of local amyloid deposits in the atrophic form of general paresis and AD, archival brain material was collected from Switzerland and Brazil from 7 demented patients (aged 42–82 years) who had clinically and neuropathologically confirmed general paresis. They all suffered from slowly progressive dementia. Paraffin sections (5 μm) from several cortical areas were stained with Bosma-Steiner and Warthin-Starry silver impregnation techniques for the demonstration of spirochetes. In addition, paraffin sections from the same cortical areas were immunostained with antibodies, which recognize several epitopes of Aβ, including Aβ 8–17 (6F/3D), Aβ17–24 (4G8), Aβ17–28 (2F9AF), Aβ40 (QCB1–40) and Aβ42 (QCB1–42, 21F12) as described earlier (Miklossy et al., 2006b). Figure 5 illustrates the results, showing the presence of beta amyloid in spirochetal colonies or spirochetal plaques, similar to immature and mature senile plaques, disseminated along the cerebral cortex, and, in some cases, in cortical and leptomeningeal vessel walls exhibiting amyloid deposits.

## Discussion

As early as 1907, Fischer proposed that senile plaques are strongly reminiscent of bacterial colonies. Recent observations claim again that senile plaques correspond to bacterial colonies (Miklossy, 1993, 2011b; Miklossy et al., 1996, 2004) and that AD is a form of chronic neurospirochetosis caused by various types of spirochetes. It was shown that similarly to *T. pallidum* various other spirochetes can also cause dementia (MacDonald and Miranda, 1987; Riviere et al., 2002; Miklossy, 2011a,b; Blanc et al., 2014). It was anticipated that if AD is caused by spirochetes, the clinical and pathological hallmarks of AD should also occur in syphilitic dementia, caused by *T. pallidum*. Therefore, through detailed historic descriptions and illustrations from the first half of the last century we verified that the clinical and pathological changes defining AD might also occur in syphilitic dementia.

Both senile plaques and spirochetal colonies were described as foci of “miliary necroses”. Senile plaques were described by Redlich (1898) as “miliary sclerosis”, by Fischer (1907) as “miliary necrosis” and Alzheimer (1907) as miliary foci of deposits of a peculiar substance.

Multiple authors described *T. pallidum* colonies confined to the cerebral cortex in general paresis (e.g., Jahnel, 1916, 1917a,b,c, 1920; Pacheco e Silva, 1926–1927) Senile plaques and spirochetal colonies are both argyrophilic, exhibit Thioflavin S fluorescence and contain beta amyloid deposition. Their localization in the cerebral cortex and their cortical laminar distribution is identical. They are confined to gray matter areas, particularly to the cerebral cortex, in the vegetative nuclei of the diencephalon and to a lesser extent in the striatum. Their presence in the white matter is rare. In the atrophic form of general paresis innumerable spirochetes accumulate without accompanying inflammatory infiltrates. Lymphoplasmocytic infiltrates are lacking, and the diffuse cortical atrophy is more accentuated in the frontotemporal regions in both AD and the atrophic form of general paresis. In both the primary motor cortex is only involved in advanced stages of the disease and in the majority of cases, the occipital lobe and cerebellum are spared. The laminar distribution of senile plaques in AD is well documented (Hof and Morrison, 1994). As noticed by Alzheimer “They tended to accumulate in the 2nd and 3rd layers, were rarer in deeper layers…”. These cortical layers with high senile plaque density are rich in capillaries (Suter et al., 2002; Miklossy, 2003). Cortical laminar distribution of spirochetal colonies (Pacheco e Silva, 1926–1927; Dieterle, 1928) and their link to the capillary network is also well known (Jahnel, 1916, 1917a,b,c, 1918, 1919, 1920, 1922; Pacheco e Silva, 1926–1927; Dieterle, 1928). Like senile plaques, they frequently accumulate in the middle cortical layers (III-IV-V), the distribution area of terminal capillaries of the short cortical arteries. Capillaries in these areas were surrounded by “dense cloud” of microorganisms.

Historic illustrations of disseminated spirochetes in general paresis, when compared to those of curly fibers in AD, provide evidence that curly fibers can correspond to individual spirochetes and their accumulation in colonies form senile plaques. Occurrence of neurofibrillary tangles (Bonfiglio, 1908; Perusini, 1910; Storm-Mathisen, 1978) was also reported in neurosyphilis. Intracellular location and proliferation of spirochetes may lead to neurofibrillary tangles and granulovacuolar degeneration as it was observed *in vitro* following exposure of primary glial and neuronal cell and organotypic cultures to spirochetes (Miklossy et al., 2006a, 2008c). Detection of spirochete specific antigens and DNA in the brains of AD patients and their localization in senile plaques, neurofibrillary tangles and granulovacuolar degeneration (MacDonald and Miranda, 1987; Miklossy, 1993; Riviere et al., 2002; Miklossy et al., 2004; Miklossy, 2011b; MacDonald, 2006) provide further evidence in support of a spirochetal origin of these structures. Argyrophylic granular forms of spirochetes similar to those occurring in AD and in silver granule dementia were also observed in the atrophic form of general paresis as illustrated by Pacheco e Silva (1926–1927).

With respect to neuronal loss and glial proliferation, Fuller (1911) noticed that the gliosis and the considerable cell loss in AD is equal in extent to the glial proliferation and cell destruction found in cases of general paresis.

The term “amyloid, was introduced in 1860 to describe “certain abnormal tissue aggregates that had staining properties similar to starch” (Torack, 1978). It is known that amyloidosis is frequently associated with chronic bacterial infections. Mignot and Marchand (1911) and later Volland (1938) defined that the “colloid degeneration” described by Alzheimer (1897) in general paresis corresponds to local amyloidosis. Recent characterization of these amyloid deposits in severe syphilitic dementia showed that, as in AD, it corresponds to Aβ (Miklossy et al., 2006b). *T. pallidum* colonies with Aβ deposits are morphologically identical to amorphous, immature and mature senile plaques. Spirochetal colonies similar to immature and mature plaques also occur in Lyme neuroborreliosis (Miklossy et al., 2004; Miklossy, 2011a) and reveal the presence of *B. burgdorferi* specific antigens, DNA and Aβ (Miklossy, 1993, 2011a; Miklossy et al., 2004). In old spirochetal colonies, morphologically identical to mature plaques, the homogenous and less argyrophilic central part also shows the presence of *B. burgdorferi* specific DNA as detected by *in situ* hybridization (see Figure 2B of Miklossy, 2011a). These observations are also in agreement with the recent observations that Aβ deposition can be induced by spirochetes *in vitro* (Miklossy et al., 2006b).

An important question is why Fischer (1907) did not find “plaques” in his 45 progressive paralysis cases analyzed. Noguchi, who together with Moore (1913) first reported the persistence of spirochetes in the brain in general paresis and detected them in 12 out of 70 general paresis cases, gives the explanation himself (Noguchi, 1914): “In the majority of cases the invasion of the pallidum takes place (or at least becomes evident) after a long period of latency (eight to twelve years from the time of syphilitic infection)”. This should be carefully considered in future studies.

The historic data presented here, strongly support recent observations showing that several types of spirochetes, in a similar way to *T. pallidum*, can cause dementia and reproduce the characteristic hallmarks of AD. The highly prevalent periodontal Treponema spirochetes, *B. burgdorferi*, intestinal spirochetes, and other invasive Borrelias and Treponemas may well disseminate and invade various organs including the brain and be responsible for the development of dementia in AD.

The helical shape of spirochetes is important for the reproduction of the characteristic filamentous pathology of senile plaques, neurofibrillary tangles and curly fibers and the atypical granular form of spirochetes, as shown *in vitro*, can lead to granulovacuolar degeneration.

Spirochetes frequently co-infect with other microorganisms (Gastinel, 1949) and *Chlamydophila pneumonia* (Balin et al., 1998, 2008; Little et al., 2014), *Porphyromonas gingivalis* (Poole et al., 2013, 2015), *Propionibacterium acnes* (Kornhuber, 1995, 1996), *Helicobacter pylori* (Kountouras et al., 2006) and herpes simplex virus type 1 (HSV-1) (Jamieson et al., 1991, 1992; Itzhaki et al., 1997; Itzhaki and Wozniak, 2008) were shown to be associated with AD. All these observations indicate that to consider infection caused by spirochetes and other microorgansims is essential. Senile plaques might correspond to biofilms and co-infections of various microorganisms would influence the evolution and increase the severity of dementia.

## Conclusion

It is established that *T. pallidum* can cause slowly progressive dementia in the atrophic form of general paresis. Recent observations showed that several types of spirochetes are involved in the etiology of AD. If AD corresponds to chronic neurospirochetoses, the clinical and pathological hallmarks of AD should also occur in syphilitic dementia. Descriptions and illustrations published in the first half of the last century indeed show that dementia, cortical atrophy associated with argyrophilic plaques, neurofibrillary tangles, likewise beta amyloid deposition are all characteristics features of the atrophic form of general paresis. These findings are historical evidence that chronic neurospirochetosis can reproduce the clinical, pathological and biological hallmarks, which define AD. They indicate that curly fibers in AD correspond to individual spirochetes, and their agglomeration in colonies produce senile plaques. These historic observations further support a causal relationship between long-standing spirochetal infections and AD and are in harmony with Alzheimer’s and Fischer’s view on the accumulation of foreign substance in senile plaques and with Fischer’s view that senile plaques are reminiscent of bacterial colonies.

## Search Strategy and Selection Criteria

The goal of search for historic literature was to answer the question whether lesions similar to AD may occur in long-standing, severe syphilitic dementia.

Therefore, we intended to search relevant literature, descriptions and illustrations on the pathological features of advanced stages of the atrophic form of general paresis, which is known to be associated with brain atrophy and severe dementia. We selected those reports where the clinical, laboratory and neuropathological examination confirmed syphilitic infection, and the histological detection of spirochetes in the brain was performed. Our first search was based on articles referenced in a chapter of Handbuch der Speziellen Pathologischen Anatomie und Histologie on the detection of spirochetes in the central nervous system in various neuro-psychiatric disorders in syphilis (Schlossberger and Brandis, 1958). Articles and books in English, French, German, Dutch, Spanish, Italian and Hungarian were all included. References of the collected articles and books served for further sources of search. Such progressive search of the relevant literature enabled us to acquire a representative number of reports and illustrations, showing the characteristic pathological features of advances stages of syphilitic dementia. Our goal was not to collect all published observations and illustrations available, but to collect high quality observations and illustrations which enable us to answer the question whether AD-like pathology occurs in syphilitic dementia and compare the pathological features of severe syphilitic dementia with those of AD. For this purpose we also collected some relevant historic literature on AD. Further search on PubMed, Google Scholar, and Science Direct, by using keywords of dementia and syphilis, yielded some additional literature, which included few recent observations on the clinical manifestation and brain atrophy in syphilis. These observations were in harmony with the historic data and were included as complementary data and references in this review. The results obtained are based on historic observations reported by others and therefore exclude any subjectivity. As mentioned in the introduction, in order exclude any partiality in the interpretations of historic data, citations for relevant subjects are frequently used.

## Contributors

MJ initiated the work and contributed to the conception, design, analysis and reproduction of the data. She wrote the manuscript, prepared illustrations and takes the responsability for the accuracy and integrity of the presented work.

## Conflict of Interest Statement

The author declares that the research was conducted in the absence of any commercial or financial relationships that could be construed as a potential conflict of interest.
